# Dataset on Catal's reagent: Sensitive detection of iron (II) sulfate using spectrophotometry

**DOI:** 10.1016/j.dib.2020.106149

**Published:** 2020-08-08

**Authors:** Funda Ozkok, Yesim Muge Sahin, Vildan Enisoglu Atalay, Kamala Asgarova, Nihal Onul, Tunc Catal

**Affiliations:** a Department of Chemistry, Istanbul University-Cerrahpasa, Avcilar, Istanbul, Turkey; b Department of Biomedical Engineering, Istanbul Arel University Turkey; c Polymer Technologies and Composite Aplication and Research Center (ArelPOTKAM), Istanbul Arel University Buyukcekmece, Istanbul, Turkey; d Istanbul Protein Research Application and Inovation Center (PROMER); e Department of Molecular Biology and Genetics, Uskudar University 34662 Uskudar, Istanbul, Turkey

**Keywords:** Catal's reagent, Iron (II) sulfate, Spectrophotometer, 1-(Dodecylthio)anthracene-9,10-dione, Thiols

## Abstract

Catal's reagent is characterized by spectroscopic methods such as fourier-transform infrared spectroscopy (FT-IR), nuclear magnetic resonance (NMR) spectroscopy, mass spectrometry (MS), ultraviolet (UV)–visible spectrophotometry. Effects of different solvents such as methanol and ethanol on absorption spectrum of 1-(Dodecylthio)anthracene-9,10-dione (3) were present. Detection range of iron (II) sulfate using Catal's reagent was analyzed. Synthesis of 1-(Dodecylthio)anthracene-9,10-dione (**3**) was explained, and absorbances of various concentrations of iron (II) sulfate (0- 10 mg mL^−1^) were measured. The possible detection mechanism was also explained. The dataset is useful to improve the detection of iron (II) sulfate in various application fields such as environmental, agricultural, sensor, food, textile and cement industries.

The study refers to: F. Ozkok, Y.M. Sahin, V. Enisoglu-Atalay, K. Asgarova, N. Onul, T. Catal, Sensitive Detection of Iron (II) Sulfate with a Novel Reagent using Spectrophotometry, Spectchim. Acta. A, 240 (2020), 118631. https://doi.org/10.1016/j.saa.2020.118631.

**Specifications table**

**Table:** 

**Subject**	Chemistry
**Specific subject area**	Analytical chemistry
**Type of data**	Table and figure
**How data were acquired**	The data were acquired: FT-IR, NMR, mass spectrometry, UV-vis spectrophotometry
**Data format**	Raw and Analyzed
**Parameters for data collection**	1-(Dodecylthio)anthracene-9,10-dione was synthesized in the laboratory.
**Description of data collection**	Catal's reagent was prepared and examined using traditional methods. The data were collected after confirmation of the structure of 1-(Dodecylthio)anthracene-9,10-dione. UV-vis spectrophotometer, FT-IR, 1H-NMR, 13C-NMR, mass spectrometer were used in the data collection.
**Data accessibility**	With the article
**Related research article**	F. Ozkok, Y.M. Sahin, V. Enisoglu-Atalay, K. Asgarova, N. Onul, T. Catal, Sensitive Detection of Iron (II) Sulfate with a Novel Reagent using Spectrophotometry, Spectrochim. Acta. A, 240 (2020), 118631. https://doi.org/10.1016/j.saa.2020.118631.

**Value of the data**

• A database of Catal's reagent is essential for characterization of 1-(Dodecylthio)anthracene-9,10-dione (3)
• The data are key for examining iron (II) sulfate in various samples.
• These data are an important reference source for research on developing novel studies to use Catal's reagent.

## 1 Data description

**Fig. 1:**
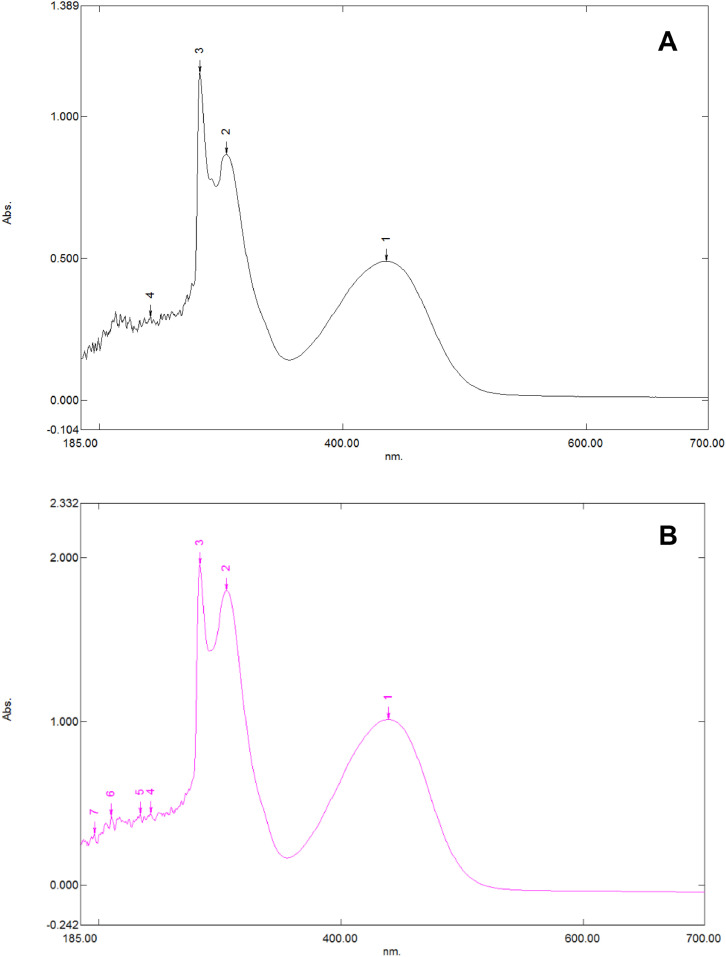
Absorption spectra of Catal's reagent in methanol (A) and ethanol (B) solution. Three similar peaks were identified.

**Fig. 2:**
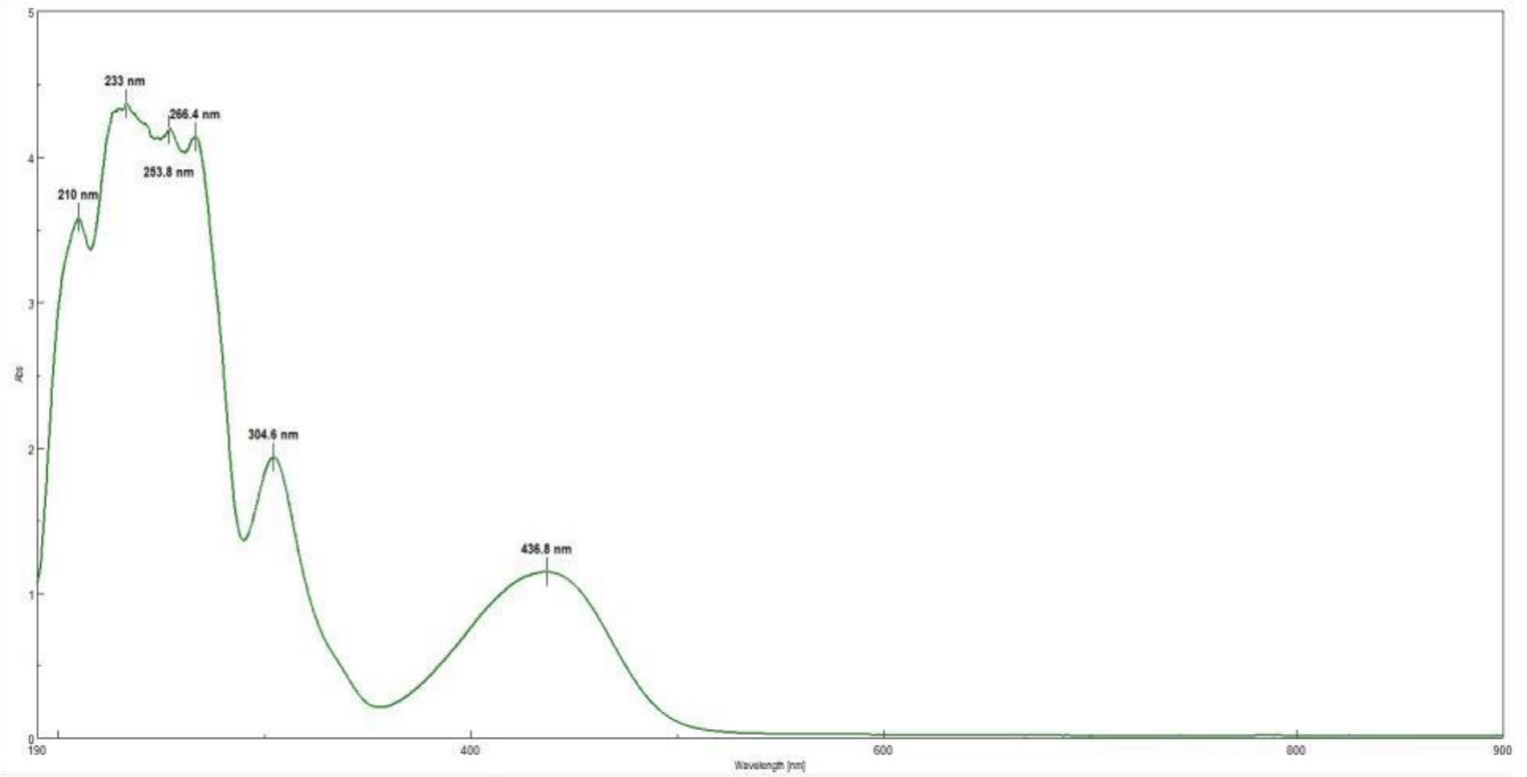
Absorption spectrum of 1-(Dodecylthio)anthracene-9,10-dione (**3**), in acetonitrile solution in lower scan rate.

**Fig. 3:**
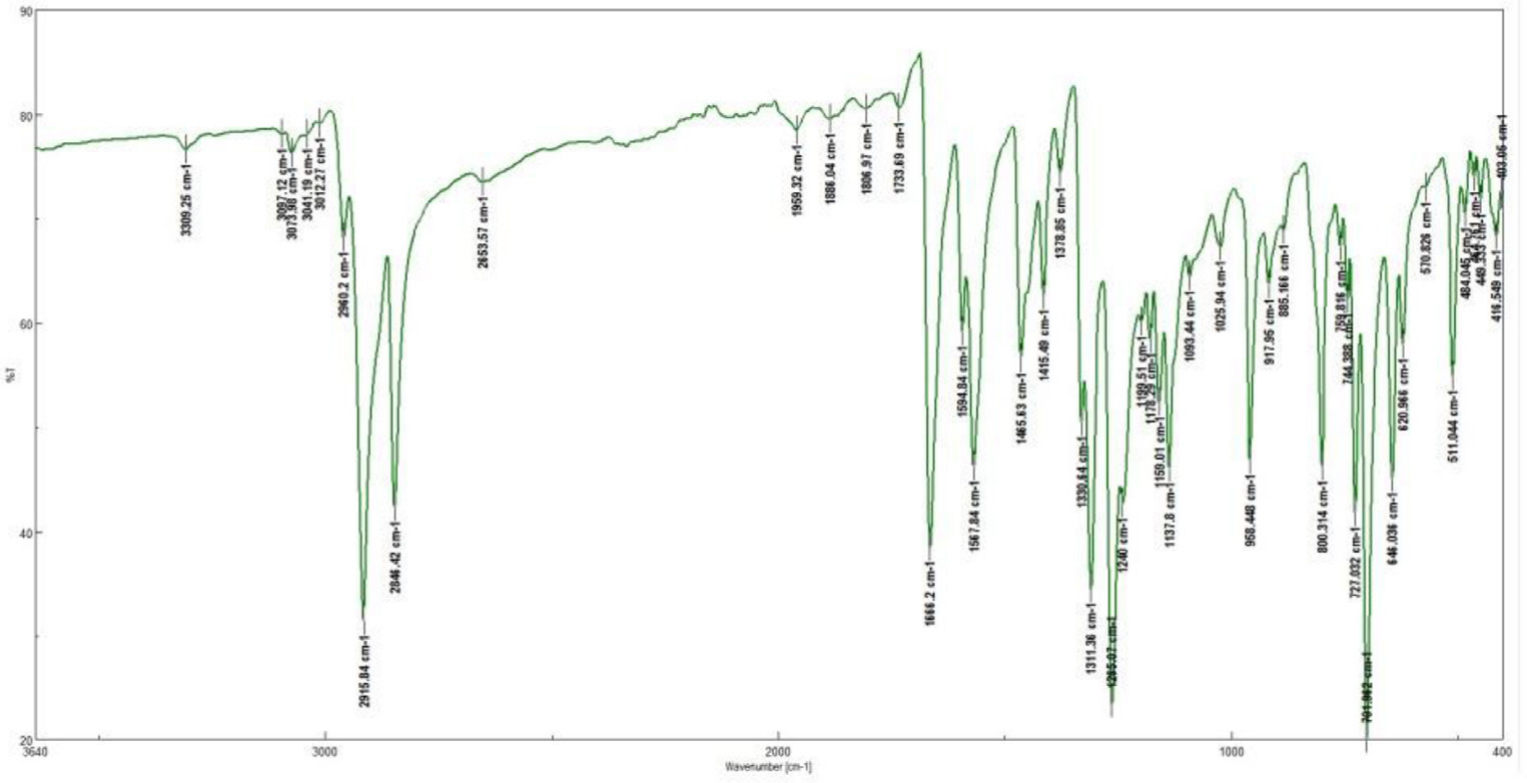
FT-IR spectrum of 1-(Dodecylthio)anthracene-9,10-dione (**3**).

**Fig. 4:**
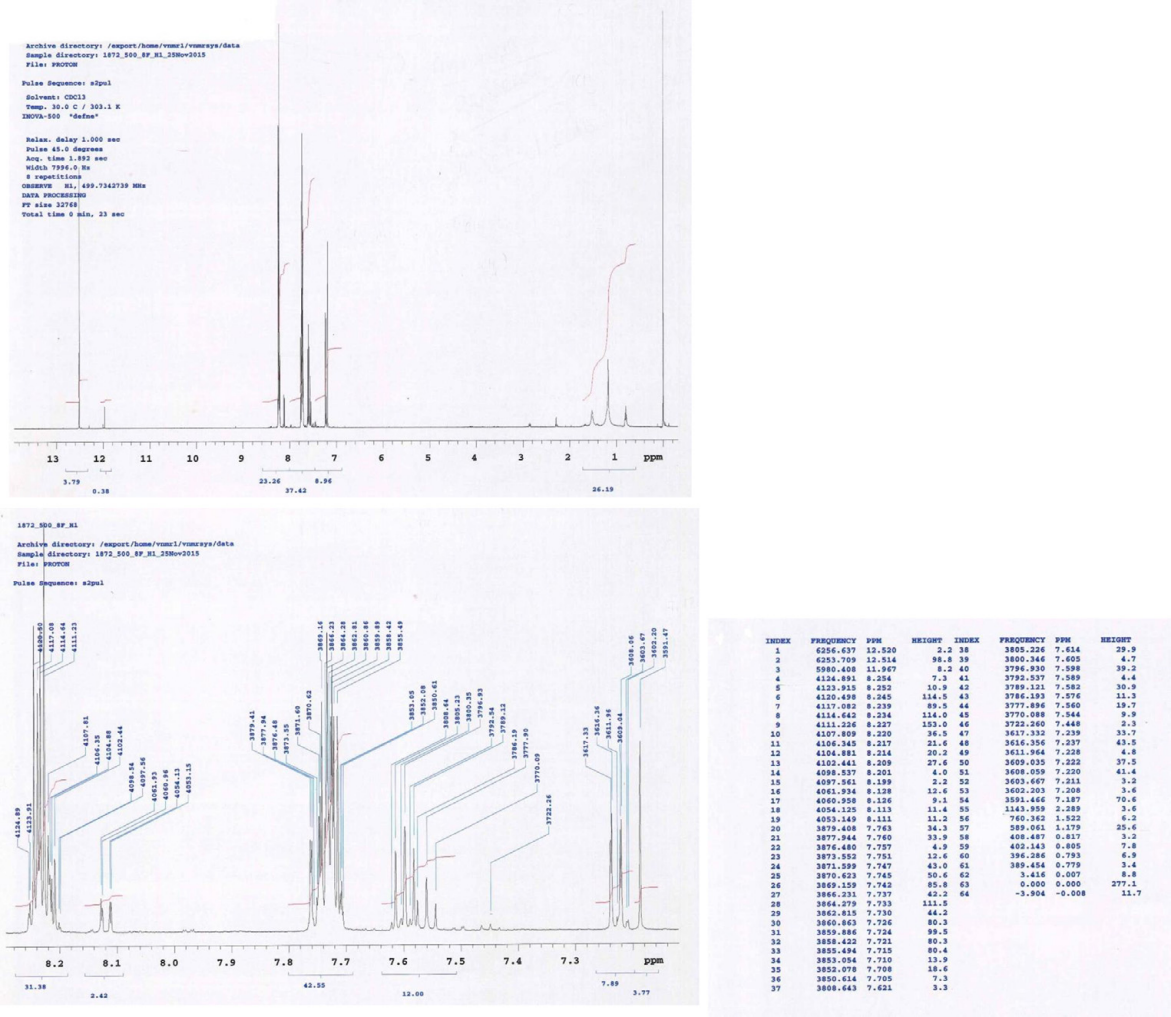
^1^H-NMR spectra of 1-(Dodecylthio)anthracene-9,10-dione (**3**).

**Fig. 5:**
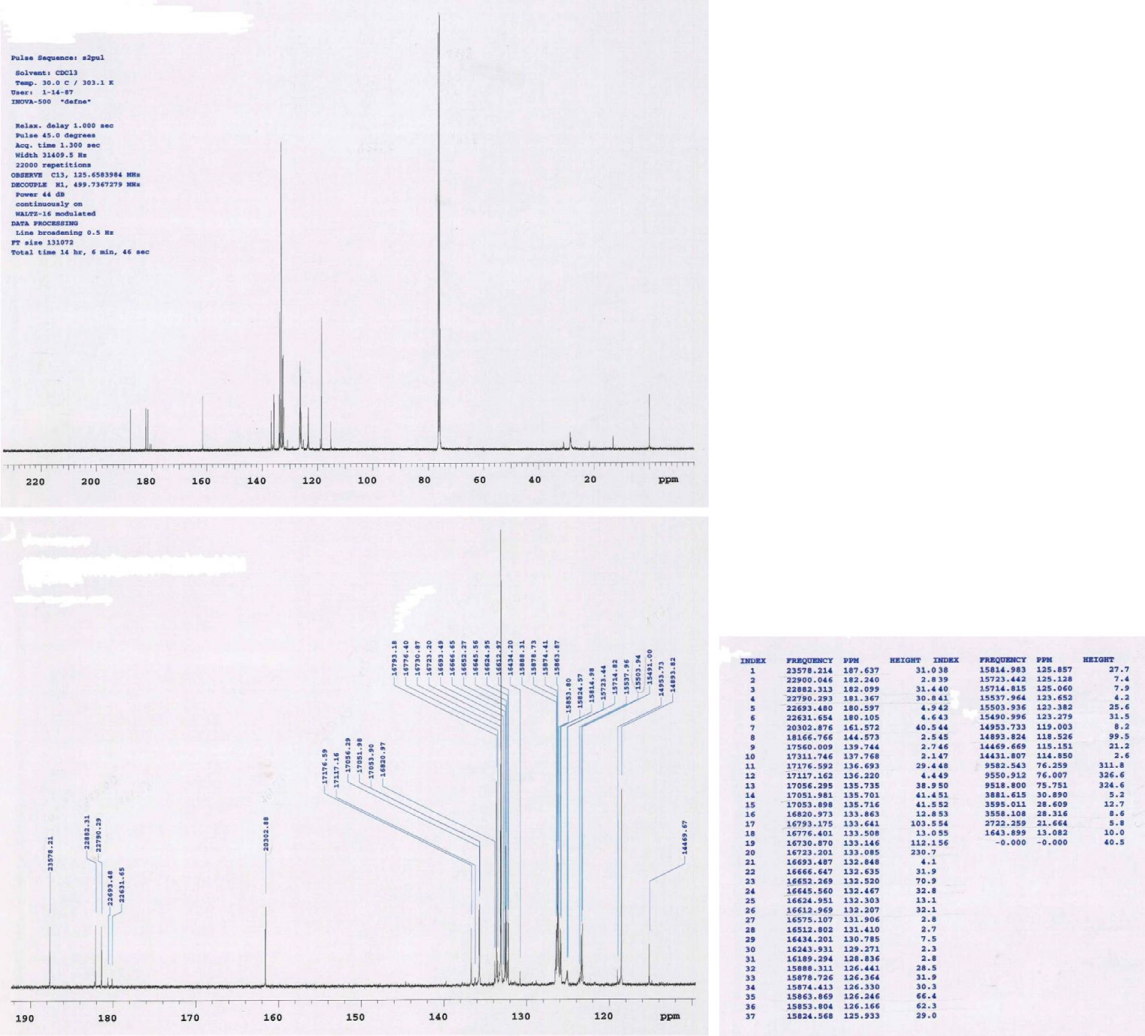
^13^C-NMR spectra of 1-(Dodecylthio)anthracene-9,10-dione (**3**).

**Fig. 6:**
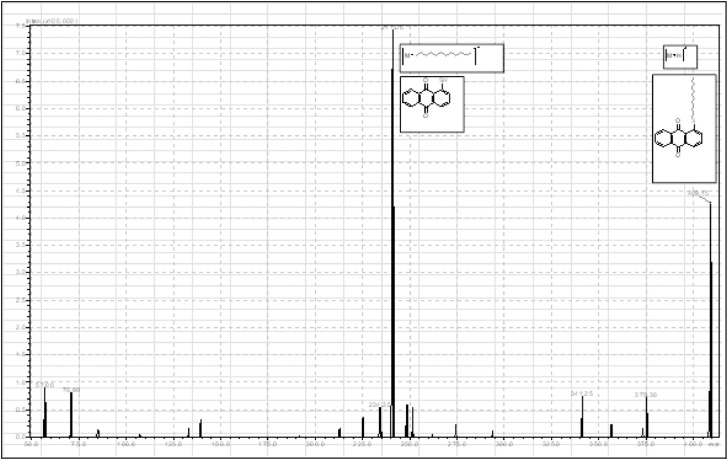
MS spectrum of 1-(Dodecylthio)anthracene-9,10-dione (**3**).

**Fig. 7:**
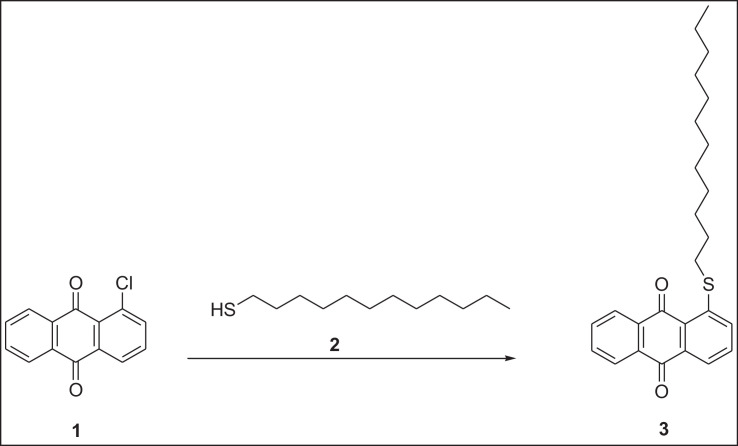
Synthesis of 1-(Dodecylthio)anthracene-9,10-dione (**3**).

**Fig. 8:**
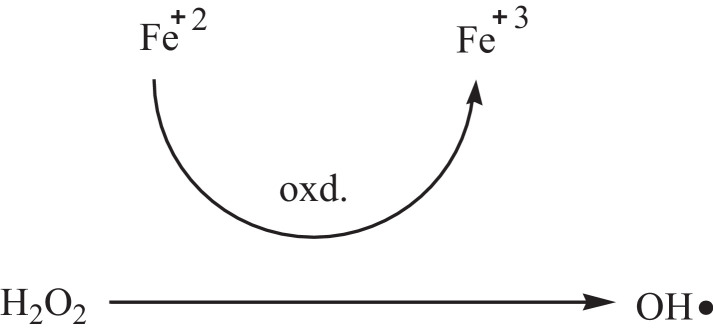
Oxidation of iron in presence of hydrogen peroxide (Fenton Reaction) [2].

**Fig. 9:**
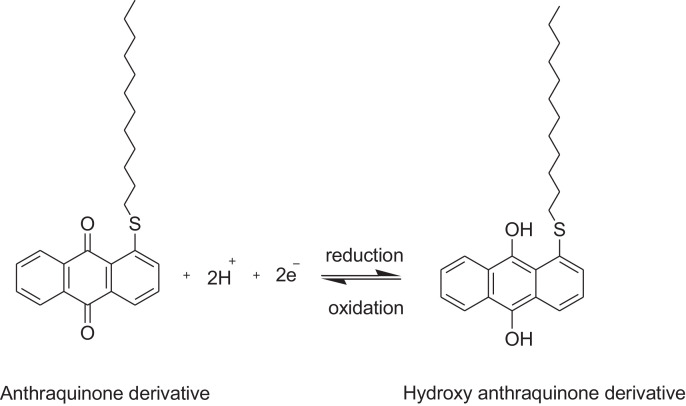
Electrochemical redox reaction of anthraquinones [3].

**Fig. 10:**
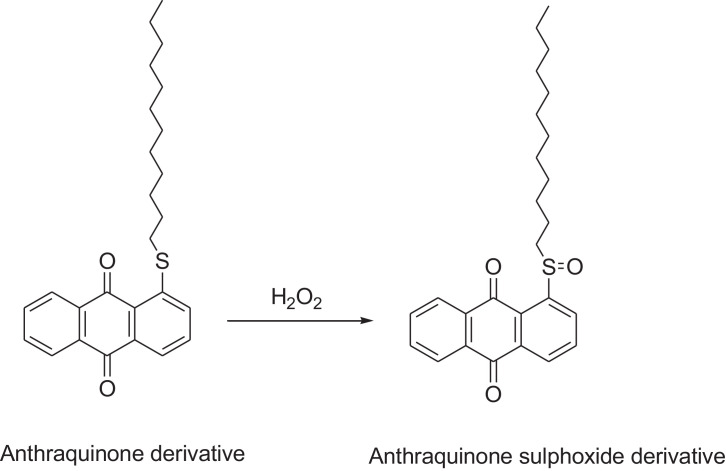
Oxidation of anthraquinone derivative in the presence of hydrogen peroxide.

**Fig. 11:**
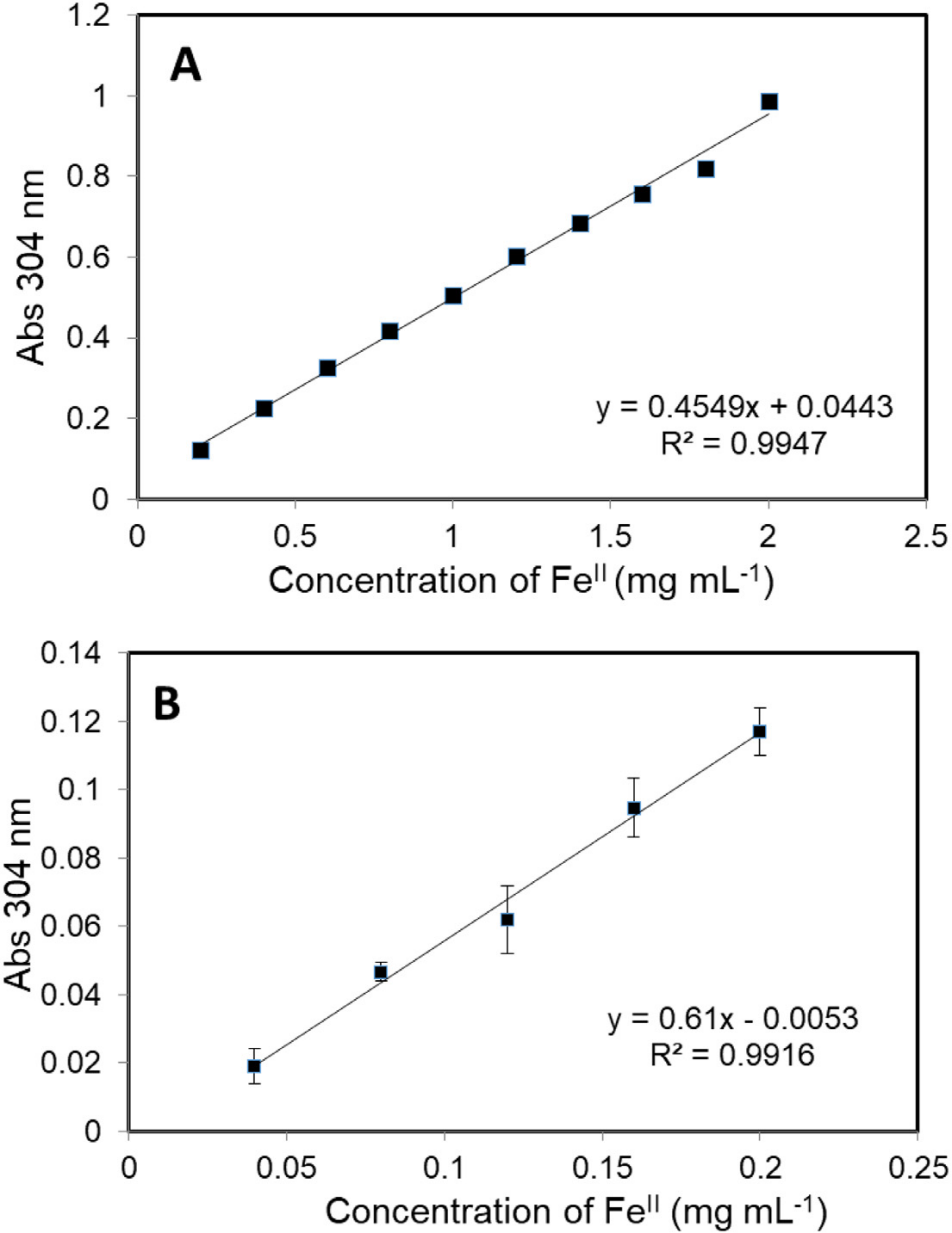
Absorbances of various concentrations of iron (II) sulfate. R^2^ values were over 0.9.

This research reports on a Catal's reagent data set for detection of iron (II) sulfate. Fig. 1 shows absorption spectra of Catal's reagent in methanol (A) and ethanol (B) solution. Fig. 2 shows absorption spectrum of 1-(Dodecylthio)anthracene-9,10-dione (3) in acetonitrile solution with lower scan rate. Fig. 3 shows FT-IR spectrum of 1-(Dodecylthio)anthracene-9,10-dione (3). Fig. 4 shows 1H-NMR spectra of 1-(Dodecylthio)anthracene-9,10-dione (3). Fig. 5 shows 13C-NMR spectra of 1-(Dodecylthio)anthracene-9,10-dione (3). Fig. 6 shows MS spectrum of 1-(Dodecylthio)anthracene-9,10-dione (3). Fig. 7 shows synthesis of 1-(Dodecylthio)anthracene-9,10-dione (3). Fig. 8 shows oxidation of iron in presence of hydrogen peroxide (Fenton Reaction). Fig. 9 shows electrochemical redox reaction of anthraquinones. Fig. 10 shows oxidation of anthraquinone derivative in the presence of hydrogen peroxide. Fig. 11 shows absorbances of various concentrations of iron (II) sulfate. Table 1 shows absorbances of several compounds at the concentration of 10 mg mL^−1^ in distilled water.

**Table 1:** Absorbances of several compounds at the concentration of 10 mg mL^−1^ in distilled water. The examined molecules were not reacted with Catal's reagent under the examined conditions.

Compound	Absorbance (304 nm)
Ammonium persulphate	-0.032
Ammonium sulphate	0.031
Aluminum potassium sulfate dodecahydrate	-0.003
Copper (III) sulphate	-0.008
Copper (III) sulphate pentahydrate	-0.056
Sodium dodecyl sulphate	-0.018
Sodium sulphate anhydrous	-0.022
Sodium thiosulphate pentahydrate	-0.002
Manganese (II) sulphate monohydrate	0.039
Magnessium sulphate heptahydrate	0.027
Sodium 2-bromoethanesulfonate hydrate	-0.029
Sulfanilic acid	0,033
Sodium sulfite anhydrous	0.001
Zinc sulphate heptahydrate	0.006
Sodium 2-chloroethane sulfonate hydrate	0.028
Potassium sulphate	0.037
Ammonium iron (III) citrate	0.012
Iron (III) citrate hydrate	0.038
Iron (II) sulfate heptahydrate	0.985

## 2 Experimental design, materials and methods

Novel thio anthraquinone derivative (1-(Dodecylthio)anthracene-9,10-dione) was synthesized by this novel method for scientific applications [1]. Chemical structure of novel thio anthraquinone compound (**3**) was characterized by spectroscopic methods such as FT-IR, NMR, MS, (UV)–visible spectrophotometry, and the structure of the compound was confirmed. The thio anthraquinone derivative, 1-(Dodecylthio)anthracene-9,10-dione (3), was dissolved in the following organic solvents to prepare the reactant named as Catal's reagent: Ethanol and methanol (Fig. 1). Tetra JASCO 6600 spectrometer used for fourier transform infrared (FT-IR) spectra, and Tetra JASCO V 750 spectrometer recorded Ultraviolet–visible spectra. A Varian UNITY INOVA at 500 MHz was used for ^1^HNMR and ^13^C NMR spectra. Mass spectra was recorded on (Shimadzu, Kyoto-Japan) LCMS-8030 triple quadrupole spectrometer in ESI (+) polarity. The absorbances were measured at 304 nM of wavelength using a UV-visible spectrophotometer in air (Shimadzu UV-2600, Cat. No. 206-27600-45, Kyoto, Japan). The reaction mixture was prepared as follow; 1-(Dodecylthio)anthracene-9,10-dione **(3)** (20 mg) was added to either ethanol, methanol or acetonitrile (60 mL) in order to prepare Catal's reagent. Catal's reagent (50 µL) was mixed with iron (II) sulfate solution (100 µL). Finally, H_2_O_2_ solution (17.5 percent in distilled water, v:v) (50 µL) was added to the mixture. 30 mM of trisodium citrate dihydrate (9 mL) was then used to stabilize pH changes. In advance, different rations of the compounds could be used to enhance the sensitivity of the reaction with Catal's reagent. Catal's reagent can be used for spectrophotometric and colorimetric detection of iron (II) sulfate [4].

## Declaration of Competing Interest

The authors (F. Ozkok, Y.M. Sahin, V. Enisoglu-Atalay, K. Asgarova, N. Onul, T. Catal) declare patent application (Turkish Patent and Trademark Office, PY2019-00552; PCT International Application, No: PCT/TR2020/050061– submitted).

## Appendix Supplementary materials

Image, application 1

Image, application 2
